# Nanochemistry of Protein-Based Delivery Agents

**DOI:** 10.3389/fchem.2016.00031

**Published:** 2016-07-20

**Authors:** Subin R. C. K. Rajendran, Chibuike C. Udenigwe, Rickey Y. Yada

**Affiliations:** ^1^Department of Environmental Sciences, Dalhousie UniversityTruro, NS, Canada; ^2^Faculty of Land and Food Systems, University of British ColumbiaVancouver, BC, Canada

**Keywords:** protein nanoparticles, bioavailability, nanochemistry, protein chemistry, nanodelivery systems

## Abstract

The past decade has seen an increased interest in the conversion of food proteins into functional biomaterials, including their use for loading and delivery of physiologically active compounds such as nutraceuticals and pharmaceuticals. Proteins possess a competitive advantage over other platforms for the development of nanodelivery systems since they are biocompatible, amphipathic, and widely available. Proteins also have unique molecular structures and diverse functional groups that can be selectively modified to alter encapsulation and release properties. A number of physical and chemical methods have been used for preparing protein nanoformulations, each based on different underlying protein chemistry. This review focuses on the chemistry of the reorganization and/or modification of proteins into functional nanostructures for delivery, from the perspective of their preparation, functionality, stability and physiological behavior.

## Introduction

The physical and chemical properties of proteins can be modified for specific food and biotechnological applications. Synthesis of protein-based nanodelivery vehicles can result in chemical and conformational changes that alter the protein functionality. Proteins have specific structures and a number of functional groups that are responsible for the physiochemical properties that can be selectively modified during the preparation of nanostructures. It is crucial to understand the underlying changes in the molecular conformation and chemical modifications induced during the production phase so as to design specific applications for these nanodelivery systems. Interfacial affinity is responsible for the adsorption of proteins as stabilizers or shells of emulsions or other materials thereby forming nanodelivery systems. In addition, the interfacial affinity of proteins for oil/water interface is greater than that of air/water interface (Santiago et al., 2008). Once the proteins are adsorbed, steric and electrostatic interactions can stabilize the particulate suspensions (Dalgleish, 2006). This review presents the different techniques employed for the production of protein nanodelivery systems from the perspective of the mechanisms involved in their formation while focusing on the conformational and chemical changes in proteins with respect to delivery applications, nanoparticle preparation, stability and cellular uptake.

## Production of protein nanodelivery systems

### Coacervation

The electrostatic attractions between various components of the nanocomplexes drive the formation of coacervated nanocomplexes. However, the protein net charge is zero at the pI, leading to pH-related instability issues for protein nanoparticles. Thus, pH plays a major role in complex formation *via* its role in determining the degree of ionization of proteins (Priftis et al., 2012). Crosslinking is one of the approaches that can be used to overcome this issue. However, Zeeb et al. (2013) demonstrated that laccase-induced crosslinking of whey protein isolate (WPI)-pectin coacervate did not improve the pH stability of the nanoparticles. Heat treatment on the other hand had better impact on the nanoparticle stability (Zeeb et al., 2013). Moreover, formation of stable WPI complexed nanoparticles has been achieved by heat induced gelation following coacervation (Dai et al., 2015). Furthermore, anionically supercharged model proteins has been used to demonstrate that higher protein charge can increase the stability of protein micelles, which were found to be resistant to heat and dehydration (Obermeyer et al., 2016). The pH-induced instability of coacervate nanocomplexes has promise for pharmaceuticals with relevance in the design of stimuli-responsive drug release particles. Even though electrostatic interactions have been implicated in coacervates associations, a contrasting mechanism has been recently reported suggesting that the enthalpy and entropic forces drive the free energy for polyion association in aqueous solutions that leads to complexation (Fu and Schlenoff, 2016). In terms of changes to secondary structure as a result of processing, it is worth mentioning that no significant complexation-induced conformational changes were observed in nanoparticles prepared from pea protein isolate and alginate (Klemmer et al., 2012).

### Heat-induced gelation

**Figure 1:**
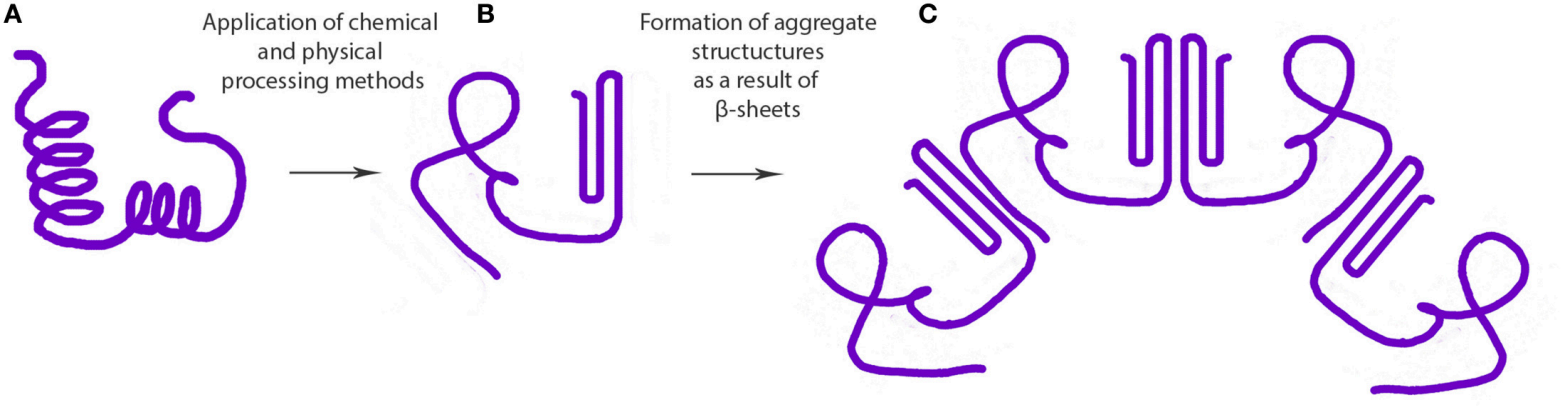
**Steps involved in the formation of protein based aggregate structures as a result of β-sheet stabilization (A) native protein, (B) misfolded or denatured proteins, and (C) supramolecular structure formed as a result of intermolecular β-sheet formation**.

Cold gelation of proteins is a two-step process involving: (1) preparation of aggregate by heat treatment, and (2) induction of gelation at ambient temperature by gradually lowering the pH (i.e., pH-induced gelation) or by the addition of salt (i.e., ionic gelation). Some of the inter- and intramolecular interactions that contribute to the process of protein gelation are ionic bonds, hydrogen bonds, hydrophobic interactions, and disulfide bonds (Sun and Arntfield, 2012). The addition of calcium or lowering the pH in the second step of the process neutralizes the structural unit surface charges leading to the formation of a three-dimensional network stabilized by some of the forces mentioned above (Maltais et al., 2008). Coacervation followed by heat-induced gelation of Maillard conjugates of WPI and dextran was found to form stable (pH 1–8, 200 mM NaCl) nanoparticles stabilized by steric repulsive and electrostatic interactions (Dai et al., 2015). Increase in temperature, protein concentration and decrease in pH were found to increase the size of ovalbumin-based nanoparticles (Sponton et al., 2015). In contrast, it was reported that thermal treatment did not improve the physical stability properties of soy protein isolate-stabilized emulsions (Fernández-Ávila et al., 2015). Moreover, heat denatured β-lactoglobulin has been used to coat and stabilize nanosuspensions (~200 nm) of paclitaxel (Li et al., 2015). Hydrophobic interactions between the protein and drug in the nanoparticle suspension resulted in the loss of both tertiary and secondary protein structures, particularly a decrease in the α-helix content (Li et al., 2015). Binding ability of proteins to polyunsaturated fatty acids (PUFA) to form nanocomplexes was also shown to depend on the surface area, hydrophobicity and spatial conformation of hydrophobic surface of the proteins (Perez et al., 2014; Sponton et al., 2015). Ohmic heating is a relatively new technique used to tailor the denaturation and aggregation behavior of proteins with the help of uniform electric fields to develop novel nano-hydrogels (Pereira et al., 2015; Rodrigues et al., 2015). This technique can introduce uniform heating facilitating the modulation of desired properties across the suspension. In general, decrease in protein structure (i.e., structured α-helix content) has been associated with heat-induced gelation. For instance, decrease in α-helix structure and increase in β-sheet/turns was reported for cruciferin nanoparticles formed *via* calcium-induced gelation (Figure 1; Akbari and Wu, 2016). Similarly, formation of calcium-induced soy protein nanoparticles also significantly increased the antiparallel β-sheet content (Zhang et al., 2012). These findings suggest a nanoparticle formation mechanism involving antiparallel β-sheets, as discussed later in this review.

### Antisolvent precipitation

**Figure 2:**
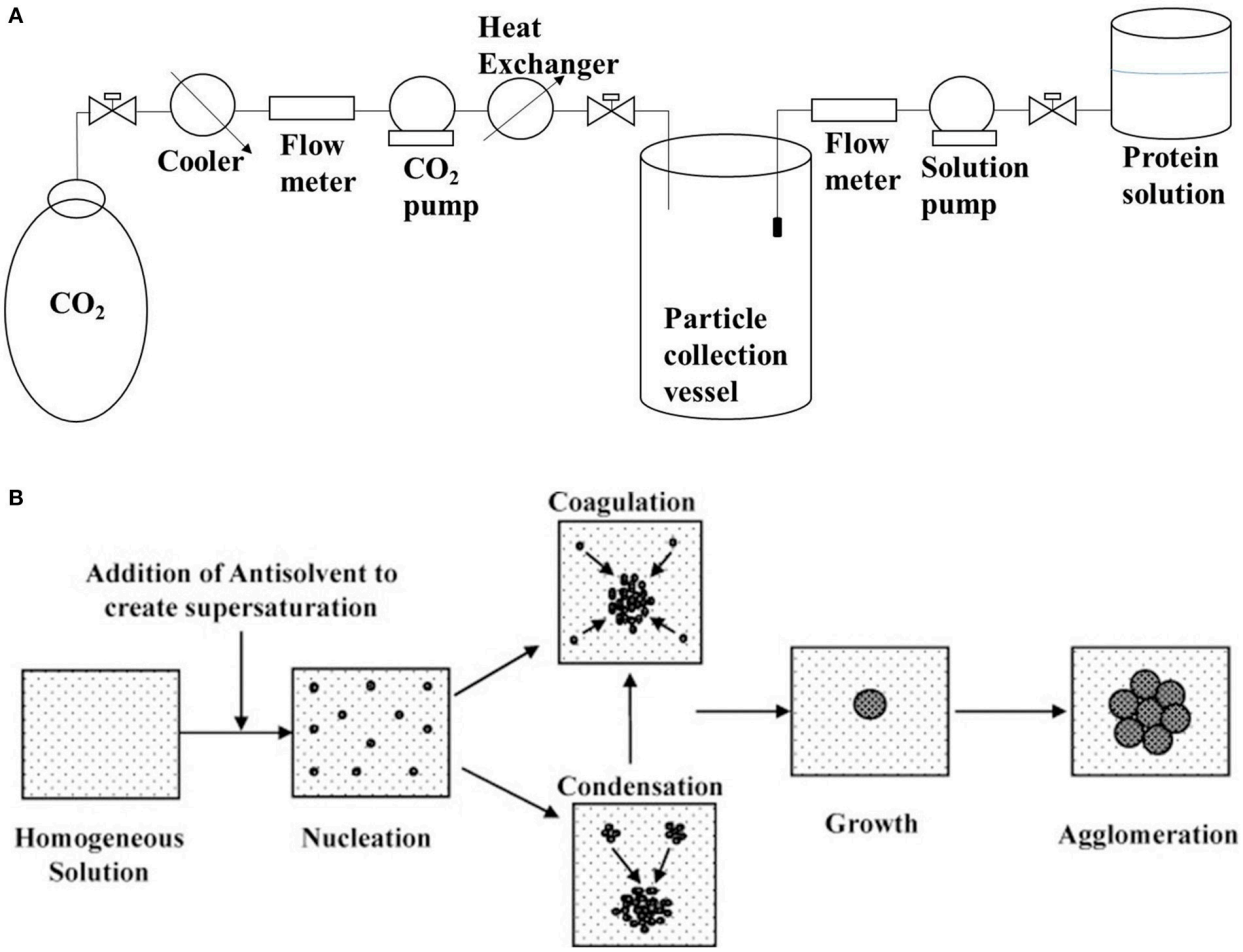
**(A)** Schematic setup of supercritical antisolvent precipitation for the synthesis of nanoparticles (based on information presented in Silva and Meireles, 2014); **(B)** Schematic of mechanism underlying liquid antisolvent precipitation (reprinted from Thorat and Dalvi, 2012 with permission from Elsevier).

Antisolvent precipitation involves the precipitation of protein aggregates by the addition of a non-solvent (antisolvent) to the protein solution, as shown in Figure 2. The major issue with solution precipitation is the solvent recovery for reuse. Supercritical fluids have been widely employed in the preparation of powder formulations of bioactive compounds because of their low cost, mild process conditions and non-flammable and non-toxic properties. Dense gas-based techniques of precipitation have been demonstrated to be suitable for the production of sub-micro biologically active protein particles with model proteins (Thiering et al., 2000). There is a consensus that the generation of uniformly sized protein micro/nanospheres can significantly promote the predictability of physiological absorption. This is evident from a study that demonstrated that salmon calcitonin prepared by supercritical fluid-assisted spray-drying has a higher absorption and bioavailability compared to spray dried and raw calcitonin (Cho et al., 2015). CO_2_-based supercritical antisolvent precipitation has also been used to prepare lysozyme nanoparticles that retained 87% of its native activity (Chattopadhyay and Gupta, 2002). Preparation of bovine serum albumin microparticles by supercritical fluid-assisted atomization, introduced by a hydrodynamic cavitation mixer (SAA-HCM), resulted in a decrease in the protein α-helix from 63% in the native protein to 55% after SAA-HCM (Wang et al., 2011). In the study, β-sheet content was found to increase by 17% with most of the secondary structure intact (Wang et al., 2011); mechanism for β-sheet increase is presented in Figure 1. Therefore, from the report on enzyme activity and conformational studies, antisolvent precipitation can be considered a relatively less destructive method. Crystallization/precipitation and evaporative precipitation are some of the other similar methods in nanoparticle synthesis that can induce protein precipitation *via* solvent unavailability.

### Enzymatic and chemical crosslinking

Chemical crosslinking is often employed to chemically harden synthesized nanoparticles by inducing inter- and intra-molecular covalent linkages. Gliadin-based nanoparticles prepared by antisolvent precipitation was found to be hardened by chemical crosslinking with glutaraldehyde, and this was demonstrated to have only a slight improvement on the stability (Joye et al., 2015). Similarly, β-lactoglobulin nanoparticles have also been chemically hardened (Teng et al., 2013, 2016). Addition of crosslinking agents has been shown to have no substantial impact on mean particle size, and it slightly decreased the zeta potential of the particles (Weber et al., 2000). To overcome the negative effects of chemical agents, such as toxicity or undesirable reactions, a recent study reported the use of ionizing radiation to prepare albumin nanoparticles by induced cross-linking (Achilli et al., 2015). However, in the study, there was a 20% loss in the α-helix content of albumin as a result of ionizing radiation treatment.

Enzymatic crosslinking has been successfully used for the preparation of protein nanoparticles of particular size and structure using transglutaminase, peroxidase, tyrosinase and laccase. Transglutaminase has been demonstrated to induce protein glycation and crosslinking, thereby improving the protein interfacial properties (Jiang and Zhao, 2010). The crosslinking process has resulted in a reduction of thickness in the adsorbed protein layer (Partanen et al., 2013), along with a decrease in ordered secondary structures (Ercili-Cura et al., 2012). However, excess crosslinking between lysine and glutamine residues can result in a decrease in protein digestibility. To address the latter, soy protein isolate deamidation has been found to decrease glycation and crosslinking induced by transglutaminase, giving the products higher digestibility, less increased hydration, lower thermal stability, and relatively unfolded secondary structure, and this can, therefore, be used as a means to control the degree of crosslinking or transglutaminase activity (Yao and Zhao, 2016).

### Ultrasonication

Ultrasonic treatment of liquids creates acoustic cavitation, which is the formation, growth and collapse of bubbles that induce localized production of heat and can drive chemical reactions. Specifically, sonolysis of water forms free radicals, •OH and H•, which can lead to the generation of superoxide anion radicals and H_2_O_2_, which in turn can act as protein crosslinking agents (Weissler, 1959). This process involves the lowering of the free protein cysteine thiol groups thereby inducing disulfide linkages. However, proteinaceous microspheres have been prepared using non-S containing streptavidin indicating that other factors also play a role in the formation of macromolecular structures besides disulfide linkages when using ultrasonication (Avivi and Gedanken, 2002). It has been demonstrated that oil-in-water emulsions prepared by sonication treatment have OH^−^ adsorbed on the outer surface that stabilizes these emulsions (Reddy and Fogler, 1980; Kamogawa et al., 2004). Whether a similar mechanism exists for protein-stabilized emulsions has yet to be elucidated. Ultrasonication can breakdown whey proteins aggregates formed during pre-heat treatment and also prevent their reformation (Ashokkumar et al., 2009). Moreover, the surface hydrophobicity of whey proteins was found to initially increase on sonication as a result of protein unfolding, with a later decrease after 5 min, presumably as a result of aggregation (Chandrapala et al., 2011). Moreover, sonication did not alter the thiol content in the study. In contrast, a study with soybean protein isolate reported an increase in thiol content following ultrasonication, and this was attributed to the exposure of SH groups in the unfolded protein (Hu et al., 2013). In contrast, ultrasound treatment of gelatin resulted in an increased solubility (Yu et al., 2016). Secondary structure modifications resulting from high-power ultrasonication of proteins differ from the effects of other treatments, with an increase in α-helix content and a decrease in β-sheet (Hu et al., 2013). However, lower power ultrasonic treatment have been demonstrated to decrease the α-helices and increase the β-sheets (Gülseren et al., 2007; Hu et al., 2013).

### High-pressure homogenization

**Figure 3:**
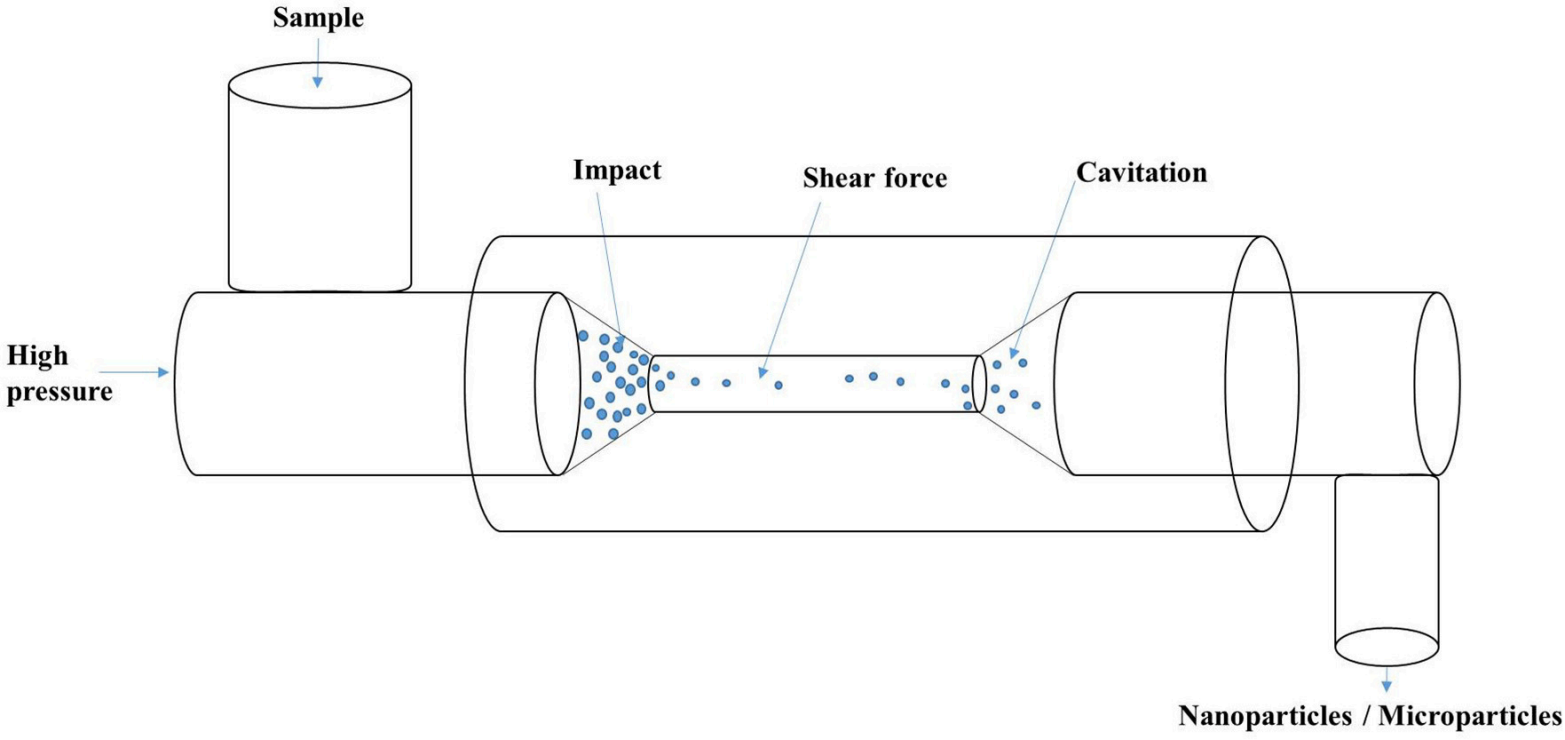
**Schematic of high-pressure homogenization showing the process of impact, shear and cavitation**.

The process of high-pressure homogenization involves the passage of a mixture through a small orifice that result in turbulent flow conditions in combination with intense shear and cavitation (Figure 3), leading to the disruption of oil droplets/protein suspensions. High-pressure homogenization (HPH) affected the supramolecular structure of soy proteins with significant increase in the denaturation temperature of treated samples (Keerati-U-Rai and Corredig, 2009). Moreover, the pressure-treated glycinin and soy protein isolate were observed to have larger soluble aggregate size, whereas β-conglycinin had a reduced aggregate amount following HPH (Keerati-U-Rai and Corredig, 2009). Dynamic high-pressure microfluidization has also been demonstrated to decrease α-helix content and increase the β-sheets of zein-derived nanoparticles (Sun et al., 2015). HPH has been demonstrated to both improve and reduce the activity of enzymes depending on the type of enzyme, pH, temperature and number of passes (Tribst et al., 2012, 2013). Therefore, HPH has potential applications in nanoencapsulation or nano-immobilization of enzymes for improved recovery and activity.

### Electrospinning

Electrospinning is a process that utilizes electrical forces to produce fibers with diameters ranging from 2 nm to several micrometers. For both electrospinning and electrospraying, a jet of liquid passes through a capillary tube, out a nozzle to a collector, and a voltage is applied between the tube and the collector. Based on the rheology of the solution emitted, the jet of liquid emitted from the nozzle either becomes dispersed as droplets or undergoes a whip-like motion to form fibers. Electrospun nanofibers have great promise in fabrication of nanostructured materials with enhanced properties. Gelatin and collagen nanofiber mats loaded with cefazolin and silver nanoparticles, respectively were demonstrated to be prospective technologies for improving wound healing (Rath et al., 2015, 2016). Nanofibers were cross-linked together using glutaraldehyde in these studies to form a mat-like structure. Moreover, zein nanofibers have been successfully fabricated for the sustained delivery of SiRNA into skin fibroblasts (Karthikeyan et al., 2015). The nature of the solvent determined the formation of either zein beads or fiber with ethonolic zein protein solutions resulting in fibers whereas isopropanolic solutions of zein generated beads (Moomand and Lim, 2014). In contrast, the extent of bead formation *via* has also been demonstrated to be controlled by the concentration of polymeric materials (Weiss et al., 2012), which in turn impacts the rheology of the solution. The extent of matrix swelling, erosion and diffusion was reported to determine the release kinetics of encapsulated fish oil from electrospun zein protein carriers (Moomand and Lim, 2014). Secondary structure modifications as a result of electrospinning can vary with protein concentration and solvent used, but a decrease in α-helical structure was evident when compared to the native protein (Moomand and Lim, 2014).

### Electrospraying

Similar to electrospinning, electrospraying is an electric field induced atomization technique that forms charged self-dispersed droplets. Electrospraying has several advantages over conventional spray drying, including the production of (1) smaller sized and narrowly distributed particles, and (2) self-dispersing charged droplets with electric field governable motion (Jaworek, 2007). Reduction of protein concentration and flow rates have been demonstrated to decrease the particle size of electrosprayed protein particles (Gomez et al., 1998). Increased production of nanoparticles can be facilitated by increased flow rate, which has resulted in larger sizes of the particles (Gomez et al., 1998). Electrospraying appears to be a relatively non-destructive method, considering the insignificant differences observed in the activity of electrosprayed and untreated insulin (Gomez et al., 1998).

### Conjugation

#### For targeted delivery

Conjugation of signaling peptides such as mitochondrial targeting signal peptides and cell-penetrating peptides has the potential to enable the targeting of nanoparticles to specific cells and cellular components. Folic acid tagged-protein nanoemulsions prepared by high-pressure homogenization was reported to promote specific folate receptor (FR)-mediated targeting FR^+^ cells (Loureiro et al., 2015). However, the effectiveness of targeted delivery with respect to non-specific protein adsorption *in vivo* needs to be characterized for protein-based nanomaterials. Some of the essential chemical strategies involving coupling, fusion and encapsulation of human serum albumin have been reviewed (Liu and Chen, 2016).

#### For enhanced stability

Proteins have almost zero charge near their pI, and this can disrupt the stabilizing effects of electrostatic interactions, and therefore, negatively affect protein stability over a range of pH. Complexation and conjugation with a number of different materials enhances the surface chemistry of the nanoparticles for improved stability and physiological effects. Dextran is a neutral α-1′ glycosidic-linked glucose polysaccharide with high solubility, low viscosity, and low gelation properties. Dextran along with pectin and chitosan has been widely applied for glycosylation of proteins to avoid the complications from the formation of electrostatic complexes. Maillard reaction conjugates of sodium caseinate and dextran coated on zein nanoparticles encapsulating resveratrol was found to significantly improved the particle stability against changes in pH (2.0–9.0), CaCl_2_ addition (100 mM) and heat treatment (30–90°C, 30 min; Davidov-Pardo et al., 2015a). Similarly, algae oil nanoemulsions stabilized by a colloidal complex of zein hydrolysate and tannic acid, a polyphenol, demonstrated increased oxidative stability and emulsifying capacity compared to nanoemulsions stabilized by the hydrolysate alone (Wang et al., 2016).

### Modifications in the secondary and tertiary structure of proteins

There is an increase in the β-sheet content in most of the chemical and physical methods employed for the synthesis of nanodelivery systems (Figure 1). These increases, in most cases, can be due to the formation of intermolecular β-sheets that stabilize protein aggregate units. Contrary to other processing methods and treatments, pulsed electric field treatments of soy protein isolate has resulted in decreased amount of β-turns, which were converted into ordered α-helix structure, thereby increasing the content of the latter (Liu et al., 2011). Along with pulsed electric fields, high power ultrasonication treatment is the only exception among processing techniques that has resulted in an increase in the organized α-helix structure (Hu et al., 2013). Proteins with increased random coil structures have been shown to have better functional properties such as emulsification and thickening as their structure allows them to bind to larger amounts of water molecules or other chemical moieties (Davidov-Pardo et al., 2015b). In addition, as mentioned earlier, complex coacervate nanoparticles of pea protein isolate and alginate did not induce significant conformational changes compared to the control (Klemmer et al., 2012).

### Conformational flexibility

Low-level oxidation in protein isolates was demonstrated to improve flexible of the tertiary structure leading to the formation of soluble aggregates with enhanced emulsion stability; higher oxidation levels have resulted in the formation of insoluble aggregates (Chen et al., 2013; Ye et al., 2013). Most theoretical predictions suggest that the improved functional properties can be due to increased protein flexibility. For instance, as peptides have greater flexibility in their structures compared to intact proteins, Phoon et al. (2014) compared the emulsifying ability of intact β-conglycinin to its protein hydrolysates in HPH nanoemulsions of fish oil. Oxidative stability of the product was improved with the hydrolysate-coated emulsions compared to intact protein at pH ≥ 7. However, uneven interfacial coverage was observed on lowering the pH along with an increase in β-sheet formation, indicating aggregation (Phoon et al., 2014). Therefore, selection of encapsulating material for nanodelivery should consider additional physiochemical properties than just their improved emulsifying ability for the development of high quality delivery systems.

### Protein interface and interactions

Numerous studies have elucidated the binding properties of bioactive compounds or drugs, and binding sites are known to increase in denatured conformation compared to the native state (Relkin et al., 2014). Understanding protein conformational changes during immobilization and adsorption can play a significant role in functional design of protein-based nanomaterials. As mentioned earlier, interfacial affinity can induce the adsorption of proteins (Santiago et al., 2008) and, depending on the properties of the encapsulating protein, it can stabilize emulsions *via* electrostatic and steric repulsions, with larger molecular weight proteins largely exhibiting both (Dalgleish, 2006). It has been demonstrated with silica nanoparticles that particle curvature plays a major role in perturbing protein secondary structure, i.e., larger diameters facilitate an increased particle-protein interaction surface thereby inducing more changes in the secondary structure (Lundqvist et al., 2004). Similarly, conformational changes at emulsion interfaces is determined by multiple factors such as inherent flexibility of the protein, the distribution of hydrophobic/hydrophilic domains within the protein sequence and the hydrophobicity of the oil phase (Zhai et al., 2013). The protein charge also plays a significant role in these interactions. Cationic β-lactoglobulin nanoparticles were found to have superior integrity compared to the native protein in simulated gastrointestinal conditions, displayed 770% higher mucoadhesion, greater transepithelial permeation, and elevated cellular uptake; the latter molecular structure displayed higher surface hydrophobicity and decrease in β-sheet conformation (Teng et al., 2013, 2016). The emulsifying properties of protein isolates are positively correlated with surface hydrophobicity, which have been found to increase upon multiple freeze–thaw cycles, and on ultrasonication (Zhang et al., 2014; Zhao et al., 2015). The relationship between β-sheet formation and the surface hydrophobicity of protein aggregate needs further studies in order to elucidate the mechanisms underlying protein nanoparticle formation.

### *In vivo* protein adsorption: relevance to targeted delivery

Rational design of applicable nanomaterials requires the establishment of the principles underlying serum protein adsorption and cellular uptake (Walkey et al., 2012). There is a consensus that the outer surface of nanomaterials within biological systems is essentially covered by a protein corona as a result of non-specific adsorption and protein interactions. Furthermore, cellular and tissue responses of the nanomaterials depend on these corona compositions, which in turn have been demonstrated to be influenced by parameters such as size, shape, temperature, pH, surface charge, surface functional groups, and hydrophilicity or hydrophobicity of the nanoparticles (Gossmann et al., 2015; Yallapu et al., 2015). Even protein corona repellant membrane-wrapped nanoparticles have resulted in a non-specific protein adsorption (corona formation). However, selective cellular uptake of these nanoparticles has been observed in antigen presenting cells (Xu et al., 2016). Furthermore, the adsorption and unfolding of a single bovine serum albumin protein was found to trigger nanoparticle aggregation (Dominguez-Medina et al., 2016). Therefore, protein adsorption and associated conformation changes can play a major role in determining nanoparticle stability.

### Release and cellular uptake

**Figure 4:**
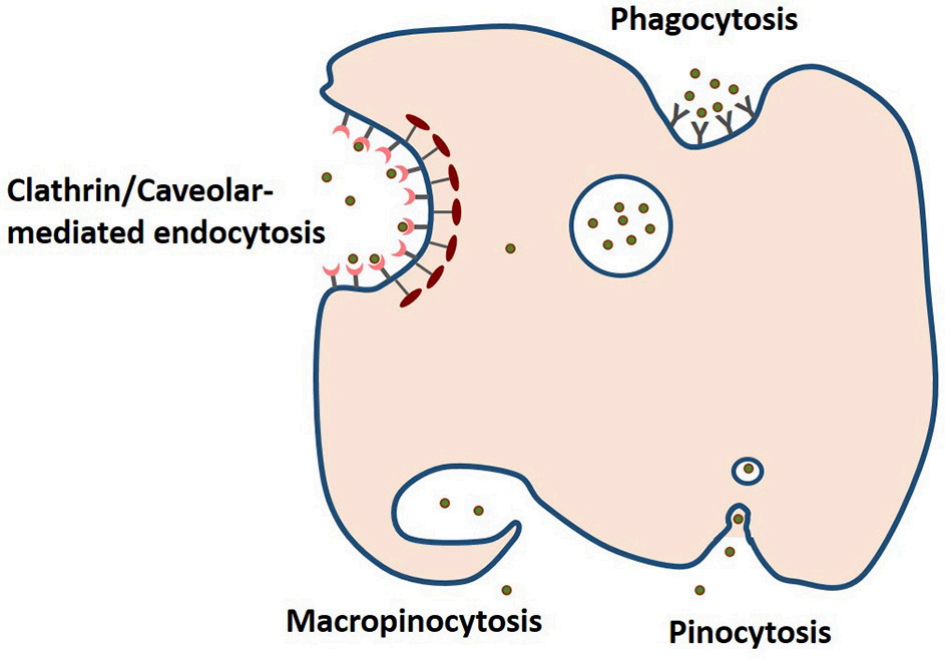
**The four pathways of cellular uptake of nanoparticules include clathrin/caveolar-mediated endocytosis, phagocytosis, macropinocytosis, and pinocytosis (based on information presented in Oh and Park, 2014)**.

Release mechanism plays an important role in determining the efficacy of bioactive compound and drug nanodelivery. As mentioned earlier, matrix swelling, erosion and diffusion determine the release of the bioactive from the carrier. For instance, resveratrol-loaded zein nanoparticles prepared by antisolvent precipitation released the polyphenol independent of pH, based on a combination of diffusion and erosion of the nanoparticle matrix leading to a 19.2-fold increase in the resveratrol oral bioavailability in a mouse model of endotoxic shock (Penalva et al., 2015). Hydrophobic bioactives are unavailable for absorption, but hydrated nanodelivery suspensions can enable them to be absorbed by a number of different mechanisms. Uptake and transport into cells depend on the stability, size, shape, ζ-potential and release of the nanoparticles. As shown in Figure 4, phagocytosis, clathrin/caveolar-mediated endocytosis, micropinocytosis and pinocytosis are some of the mechanisms for cellular uptake of nanoparticles (Oh and Park, 2014). For instance, transferrin-coated Au particles (< 50 nm) were reported to enter cells *via* clathrin-mediated endocytosis pathway. In contrast, β-lactoglobulin-stabilized paclitaxel nanosuspensions were demonstrated to directly translocate across the membrane into the cytosol in an energy-independent manner, i.e., *via* cholesterol-dependent membrane-fusion process (Li et al., 2015). Moreover, the shape of these nanomaterials can also influence this mode of cellular uptake. Cylindrical shaped nanoparticles are proposed to penetrate across the plasma membrane similar to a nanosyringe (Li et al., 2015). Surface-exposed functional groups can also determine the efficiency of cellular uptake of nanoparticles even under the influence of protein corona (Bai et al., 2015). Modification and masking of these surface functional groups will enable the tailoring of cellular uptake behavior of nanoparticles. To date, the major modes of cellular uptake of protein-encapsulated bioactive intended for food applications have yet to be determined.

## Conclusions and future direction

Interplay of the protein conformational flexibility, surface hydrophobicity, net charge and many inter- and intra-molecular interactions underlie the mechanisms of encapsulation with respect to nanodelivery. The potency of protein-based nanodelivery systems for targeted delivery of bioactive compounds is also highly dependent on the release mechanism and cellular uptake. There is still the need to elucidate the influence of preparation methods on the conformational state of proteins for nanodelivery. Several proteins from diverse sources have been used for the preparation of nanodelivery systems. However, there is a dearth of information on the physiochemical and biological properties of each of these proteins particularly related to their effectiveness as nanocarriers. Furthermore, the impact of carrier matrices such as oils (for delivery of hydrophobic bioactives) needs further consideration. *In vivo* protein corona can influence targeted delivery, but this has yet to be characterized for protein-based nanoparticles from the perspective of delivery efficiency. Filling some of these gaps is a major stride toward understanding and developing effective protein-based delivery systems for food applications.

## Author contributions

SR, CU, and RY participated in the conception of the work. SR conducted the literature search and drafted the manuscript. CU and RY revised the scientific content of the manuscript.

### Conflict of interest statement

The authors declare that the research was conducted in the absence of any commercial or financial relationships that could be construed as a potential conflict of interest.
